# Supplementary material for: Roughing it: terrain is crucial in identifying novel translocation sites for the vulnerable brush-tailed rock-wallaby (Petrogale pencillata)
Source: R Soc Open Sci. 2020 Dec 23;7(12):201603. doi: 10.1098/rsos.201603 (PMC7813239; doi:10.1098/rsos.201603)
Supplement: Figure S1 [file rsos201603supp1.docx]

| **Habitat type in this study** | **The National Vegetation Information System (NVIS) Version 5.1 for Major Vegetation Groups [1]** |
| --- | --- |
| **Woodland/forests** | 1 Rainforests and Vine Thickets |
|  | 2 Eucalypt Tall Open Forests |
|  | 3 Eucalypt Open Forests |
|  | 4 Eucalypt Low Open Forests |
|  | 5 Eucalypt Woodlands |
|  | 6 Acacia Forests and Woodlands |
|  | 7 Callitris Forests and Woodlands |
|  | 8 Casuarina Forests and Woodlands |
|  | 9 Melaleuca Forests and Woodlands |
|  | 10 Other Forests and Woodlands |
|  | 11 Eucalypt Open Woodlands |
|  | 12 Tropical Eucalypt Woodlands/Grasslands |
|  | 13 Acacia Open Woodlands |
|  | 14 Mallee Woodlands and Shrublands |
|  | 15 Low Closed Forests and Tall Closed Shrublands |
|  | 30 Unclassified Forest |
|  | 31 Other Open Woodlands |
|  | 32 Mallee Open Woodlands and Sparse Mallee Shrublands |
| **Grassland/shrublands/sedgelands** | 16 Acacia Shrublands |
|  | 17 Other Shrublands |
|  | 18 Heathlands |
|  | 19 Tussock Grasslands |
|  | 20 Hummock Grasslands |
|  | 21 Other Grasslands, Herblands, Sedgelands and Rushlands |
|  | 22 Chenopod Shrublands, Samphire Shrublands and Forblands |
|  | 26 Unclassified Native Vegetation |
|  | 27 Naturally Bare - sand, rock, claypan, mudflat |
|  | 29 Regrowth, Modified Native Vegetation |

**Figure S1**. The breakdown of the woodland and grassland variables.

**References**

1 Department of Environment. Australia—Present Major Vegetation Groups—NVIS Version 5.1 (Albers 100 m analysis product). 2012.
